# Сниженный уровень окситоцина в крови при ожирении и у пациентов с недавно диагностированным диабетом 2 типа

**DOI:** 10.14341/probl13587

**Published:** 2026-05-20

**Authors:** С. Ш. Анварова, Г. Д. Наримова

**Affiliations:** Республиканский специализированный научно-практический центр эндокринологии им. акад. Туракулова Я.Х.; Republican Specialized Scientific and Practical Center of Endocrinology named after Acad. Y.H. Turakulov

**Keywords:** окситоцин, ожирение, сахарный диабет 2 типа, инсулинорезистентность, oxytocin, obesity, type 2 diabetes, insulin resistance

## Abstract

**ОБОСНОВАНИЕ:**

ОБОСНОВАНИЕ. Ожирение и сахарный диабет 2 типа (СД2) являются широко распространенными метаболическими нарушениями. Окситоцин, известный нейропептид, в последние годы рассматривается как потенциальный регулятор энергетического обмена, аппетита и чувствительности к инсулину.

**ЦЕЛЬ:**

ЦЕЛЬ. Изучить взаимосвязь между уровнями окситоцина в крови и метаболическими показателями у пациентов с ожирением и недавно диагностированным СД2.

**МАТЕРИАЛЫ И МЕТОДЫ:**

МАТЕРИАЛЫ И МЕТОДЫ. В исследование включено 96 человек, разделенных на группы с нормальной толерантностью к глюкозе (НТГ, n=48) и с впервые выявленным СД2 (n=48). Каждая группа подразделялась на подгруппы с нормальным весом и ожирением. Измерялись антропометрические параметры, показатели углеводного и липидного обменов, инсулин, гликированный гемоглобин (HbA1c), а также высокочувствительный С-реактивный белок. Инсулинорезистентность и функцию β-клеток оценивали с помощью модели HOMA.

**РЕЗУЛЬТАТЫ:**

РЕЗУЛЬТАТЫ. Уровни окситоцина были значительно ниже у пациентов с СД2 по сравнению с пацентами с НТГ (p<0,01), а также у лиц с ожирением по сравнению с имеющими нормальную массу тела (p<0,01). Низкий окситоцин отрицательно коррелировал с ИМТ, окружностью талии, HbA1c, глюкозой, инсулином, липидами и вч-СРБ, но положительно с функцией β-клеток. Множественная регрессия показала, что уровень глюкозы через 2 часа, ИМТ и общий холестерин являются независимыми предикторами снижения окситоцина.

**ЗАКЛЮЧЕНИЕ:**

ЗАКЛЮЧЕНИЕ. У пациентов с ожирением и ранним СД2 выявлено значительное снижение циркулирующего окситоцина, ассоциированное с неблагоприятными метаболическими профилями. Эти результаты подчеркивают возможную роль окситоцина как биомаркера ранней метаболической дерегуляции и открывают перспективы для разработки новых терапевтических подходов.

## Abstract (en)

**BACKGROUND:**

BACKGROUND. Obesity and type 2 diabetes (T2D) are common metabolic disorders. Oxytocin, a hypothalamic neuropeptide, has recently been recognized as a potential regulator of energy balance, appetite, and insulin sensitivity.

**OBJECTIVE:**

OBJECTIVE. To investigate the relationship between circulating oxytocin levels and metabolic parameters in individuals with obesity and newly diagnosed T2D.

**MATERIALS AND METHODS:**

MATERIALS AND METHODS. A total of 96 participants were enrolled, including 48 with normal glucose tolerance (NGT) and 48 with newly diagnosed T2D, each subdivided into normal-weight and obese groups. Anthropometric indices, glucose and lipid parameters, insulin, HbA1c, and high-sensitivity C-reactive protein were measured. Insulin resistance and β-cell function were assessed using the HOMA model.

**RESULTS:**

RESULTS. Circulating oxytocin levels were significantly lower in patients with T2D compared to NGT (p < 0.01), and in obese compared to normal-weight participants (p < 0.01). Low oxytocin was negatively correlated with BMI, waist circumference, HbA1c, glucose, insulin, lipids, and hs-CRP, but positively with β-cell function. Multiple regression analysis identified 2-hour glucose, BMI, and total cholesterol as independent predictors of oxytocin levels.

**CONCLUSION:**

CONCLUSION. Patients with obesity and newly diagnosed T2D exhibit markedly reduced circulating oxytocin levels, associated with adverse metabolic profiles. These findings highlight oxytocin as a potential biomarker of early metabolic dysregulation and suggest new therapeutic perspectives.

## ВВЕДЕНИЕ

Ожирение и сахарный диабет 2 типа (СД2) являются одними из ведущих глобальных проблем здравоохранения, ассоциированных с ростом заболеваемости, преждевременной смертностью и существенной нагрузкой на систему здравоохранения [1][2]. Патогенез этих состояний включает сложное взаимодействие генетических, поведенческих и экологических факторов, однако все больше данных указывает на важную роль нейроэндокринных регуляторов в поддержании энергетического баланса и гомеостаза глюкозы.

Окситоцин — нейропептид гипоталамуса, традиционно известный своей ролью в родах и лактации, — в последние годы привлекает внимание исследователей благодаря более широким физиологическим функциям, включая регуляцию социального поведения, стресса и когнитивных процессов [3–6]. За пределами центральной нервной системы окситоцин оказывает значимые периферические эффекты, влияя на метаболизм. Экспериментальные исследования показали, что введение окситоцина снижает потребление пищи, усиливает липолиз, улучшает чувствительность к инсулину и уменьшает проявления ожирения у животных [7–12].

Клинические наблюдения у человека также свидетельствуют о том, что окситоцин может играть роль в регуляции метаболизма глюкозы, липидного обмена и массы тела [13–16]. В частности, недавние работы показали, что интраназальное введение окситоцина способствует снижению аппетита, уменьшению массы тела и улучшению показателей углеводного обмена у пациентов с ожирением [17–19]. Кроме того, новые данные указывают, что окситоцин может оказывать влияние на β-клеточную функцию и толерантность к глюкозе у человека [20]. Тем не менее сведения о циркулирующем уровне окситоцина при метаболических нарушениях остаются ограниченными и противоречивыми [21].

Особенно мало изучен уровень окситоцина у пациентов с впервые выявленным СД2, ранее нелеченным СД2, а также его связь с маркёрами метаболического риска. Кроме того, большая часть клинических исследований выполнена в западных популяциях, что ограничивает возможность экстраполяции данных на другие этнические группы.

В этой связи цель настоящего исследования заключалась в оценке связи между уровнем циркулирующего окситоцина и антропометрическими, биохимическими и воспалительными параметрами у лиц с ожирением и впервые выявленным СД2. Фокус на центральноазиатской выборке позволяет внести новый вклад в понимание потенциальной роли окситоцина как биомаркера ранней метаболической дерегуляции.

## МАТЕРИАЛЫ И МЕТОДЫ

В исследование было включено 96 участников в возрасте 30–60 лет (рис. 1). Участники были разделены на две основные группы:

1) лица с нормальной толерантностью к глюкозе (НТГ) — n=48;

2) пациенты с впервые выявленным СД2 — n=48.

**Figure:**
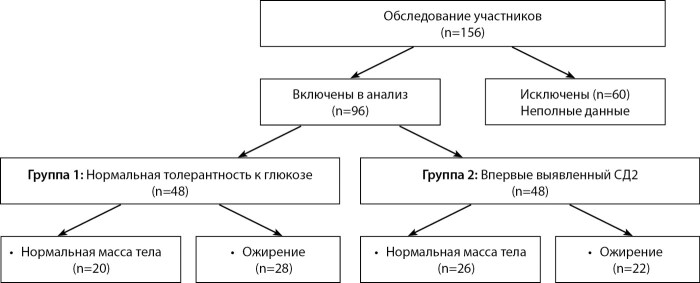
Рисунок 1. Блок-схема распределения участников исследования по группам и подгруппам (CONSORT-диаграмма).

Каждая из указанных групп дополнительно стратифицирована по индексу массы тела (ИМТ):

Таким образом, в исследовании анализировались четыре подгруппы:

Контрольную группу составили лица с нормальной толерантностью к глюкозе (НТГ), среди которых были как участники с нормальной массой тела, так и с ожирением (n=20 и n=28 соответственно).

Критерии включения: возраст 30–60 лет, согласие на участие, отсутствие предшествующей сахароснижающей терапии.

Критерии невключения: беременность, острые и хронические воспалительные заболевания, тяжелая соматическая патология, психические расстройства, прием глюкокортикоидов.

Критерии исключения: отказ от участия, неполные данные лабораторных или антропометрических исследований.

Протокол исследования был одобрен локальным этическим комитетом Республиканского специализированного научно-практического медицинского центра эндокринологии им. акад. Я.Х. Туракулова (№ 1/2023, 17.01.2023). Все участники подписали информированное согласие. Исследование проводилось в соответствии с Хельсинкской декларацией.

Концентрацию окситоцина в сыворотке крови определяли методом конкурентного иммуноферментного анализа (Competitive-ELISA) с использованием коммерческого набора Elabscience® OT (Oxytocin) ELISA Kit (Cat. No: E-EL-0029, Elabscience, Китай). Чувствительность метода составляла 9,38 пг/мл, рабочий диапазон измерений — 15,63–1000 пг/мл. Внутрисерийный и межсерийный коэффициенты вариации были <10%, что соответствует заявленным характеристикам набора.

Перед анализом все образцы сыворотки были разморожены однократно, центрифугированы при 1500 g в течение 10 минут при +4 °C для удаления осадка. Пробы исследовали в дупликатах, результаты рассчитывали на основании стандартной кривой, построенной методом четырехпараметрической логистической регрессии.

Оценивались масса тела, рост, окружность талии и бедер, рассчитывались ИМТ и отношение талии к бедрам (ОТ/ОБ). Уровни глюкозы натощак и через 2 часа, инсулина, HbA1c, липидного профиля и высокочувствительного С-реактивного белка (вч-СРБ) определялись стандартными методами. Инсулинорезистентность и функция β-клеток рассчитывались с использованием модели HOMA (HOMA-IR и HOMA-β).

Данные анализировались с использованием программы SPSS v28 (IBM, США) и R 4.3.2 (пакеты stats, car, pROC). Нормальность распределения оценивалась тестом Шапиро–Уилка. Непараметрические данные лог-трансформировались. Для сравнения групп использовались:

Для учета множественных сравнений применялся метод контроля ложного открытия (Benjamini–Hochberg FDR, q=0,05). В таблицах и рисунках приводились как неисправленные p, так и q-значения.

Множественная линейная регрессия использовалась для выявления независимых предикторов уровня окситоцина. В модель включались переменные, определенные a priori (возраст, пол, ИМТ, постпрандиальная гликемия, общий холестерин). Для оценки коллинеарности рассчитывали VIF (<5). Логистическая регрессия применялась для анализа риска СД2 в зависимости от тертилей окситоцина. Адекватность моделей оценивалась по критериям Hosmer–Lemeshow и площади под ROC-кривой.

Доля пропущенных данных для ключевых переменных составила <5%. Анализ проводился по принципу complete case. Выбросы определялись как значения за пределами ±4 стандартных отклонений или физиологически невозможные, и проверялись на предмет влияния на результаты с помощью чувствительного анализа.

## Соответствие нормам биомедицинской этики

Проведенное исследование соответствует действующим нормам биомедицинской этики, установленными международными (Хельсинкская декларация Всемирной медицинской ассоциации, 2013 г.) и национальными нормативными документами Республики Узбекистан.

Протокол исследования был рассмотрен и одобрен этическим комитетом при Республиканском специализированном научно-практическом медицинском центре эндокринологии им. академика Ю.Х. Туракулова Министерства здравоохранения Республики Узбекистан. Решение об одобрении было оформлено протоколом №1 от 17.01.2023. Все участники были ознакомлены с условиями участия и предоставили письменное информированное согласие до включения в исследование.

## РЕЗУЛЬТАТЫ

В исследование были включены 96 пациентов, разделенных на группы с ожирением (n=48) и с впервые выявленным СД2 (n=48). Исходные клинико-антропометрические и биохимические характеристики участников представлены в таблице 1.

**Table:** Таблица 1. Антропометрические характеристики и метаболические показатели (данные представлены как M ± SD)

Показатель	НТГ(n=48)	СД2(n=48)	p-значение
Возраст (лет)	45,0±9,0	48,0±8,0	0,088
Рост (см)	175,0±8,0	165,0±7,0	<0,0001
Вес (кг)	83,9±12,4	66,7±10,3	<0,0001
ИМТ (кг/м²)	35,3±4,2	33,7±3,8	0,053
HbA1c (%)	5,3±0,3	5,2±0,3	0,106
Глюкоза натощак (ммоль/л)	5,4±0,4	5,± 0,5	<0,0001
Постпрандиальная гликемия (ммоль/л)	6,0±1,2	5,7±1,1	0,205
Инсулин натощак (мЕд/л)	15,3±3,5	12,7±2,8	<0,0001
Постпрандиальный инсулин (мЕд/л)	29,0±15,0	24,0±12,0	0,075
Триглицериды (ммоль/л)	1,5±0,8	1,2±0,6	0,024
Общий холестерин (ммоль/л)	5,2±0,7	5,0±0,6	0,197
Холестерин ЛПВП (ммоль/л)	1,2±0,3	1,5±0,3	<0,0001
Холестерин ЛПНП (ммоль/л)	3,4±0,6	3,0±0,5	0,001
Окситоцин (нг/л)	9,2±1,1	9,9±1,7	0,058

## Межгрупповые различия

У пациентов с ожирением по сравнению с группой СД2 отмечались более высокие значения ИМТ (p<0,001), окружности талии (p<0,001), соотношения талия/бедро (p=0,006) и уровня инсулина натощак (p<0,001). По липидному профилю выявлены более низкие уровни холестерина ЛПВП (p=0,002) и тенденция к повышению холестерина ЛПНП (p=0,077) и триглицеридов (p=0,070). Индексы HOMA-IR (p=0,002) и HOMA-β (p=0,001), а также уровень высокочувствительного С-реактивного белка (вч-СРБ, p=0,001) были достоверно выше в группе ожирения. Уровень окситоцина оказался значительно ниже у пациентов с ожирением по сравнению с больными СД2 (p=0,003). Различий по возрасту, HbA1c, гликемии натощак и постпрандиальной, а также по общему холестерину между группами не выявлено (p>0,05).

## Визуализация межгрупповых различий

На heatmap p-значений (рис. 2) наиболее выраженные различия продемонстрированы для антропометрических показателей, уровней инсулина, HOMA-индексов, вч-СРБ и окситоцина.

**Figure:**
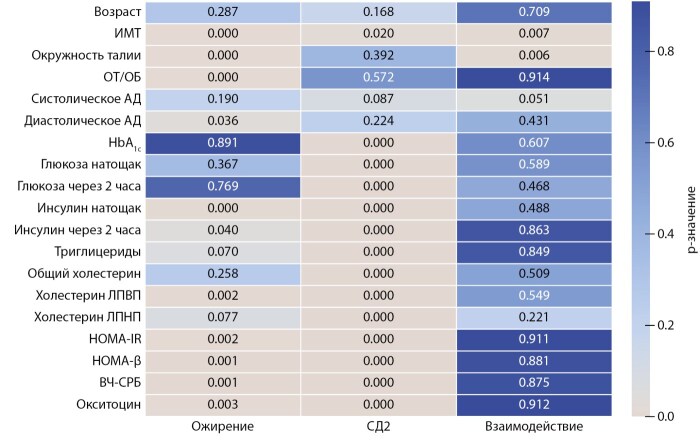
Рисунок 2. Heatmap p-значений межгрупповых сравнений (ожирение vs СД2) и их взаимодействия. Более темный цвет соответствует более высоким p-значениям. Примечания: темно-синий цвет указывает на статистически значимые различия (p<0,05), красный и светло-синий цвета обозначают отсутствие значимых различий (p≥0,05), средний тон между ними (ближе к белому) означает приближение к пороговому уровню значимости.

## Характеристики по тертилям окситоцина

При стратификации по тертилям уровня окситоцина (табл. 2) участники верхнего тертиля имели достоверно более низкие значения ИМТ, окружности талии, соотношения талия/бедро, HbA1c, гликемии натощак и постпрандиальной, уровня инсулина (натощак и постпрандиальный), триглицеридов, общего холестерина, ЛПНП-Х, HOMA-IR и вч-СРБ по сравнению с участниками среднего и нижнего тертилей (p<0,05 или p<0,01). Для ХЛВП и HOMA-β отмечалась тенденция к более высоким значениям в верхнем тертиле, однако различия не достигли статистической значимости. Распределение параметров по тертилям показано на рисунке 3.

**Table:** Таблица 2. Результаты дисперсионного анализа (ANOVA) по группам «Ожирение» и «СД2» с оценкой взаимодействия

Параметры	df	F(Oжирение)	p	df	F(СД2)	p	df	FВзаимодействие	p
Возраст, лет	1	1,143	0,287	1	1,918	0,168	1	0,140	0,709
ИМТ, кг/м²	1	183,290	<0,001	1	5,478	0,020	1	7,411	0,007
Окружность талии, см	1	154,784	<0,001	1	0,738	0,392	1	7,626	0,006
ОТ/ОБ	1	27,301	<0,001	1	0,320	0,572	1	0,012	0,914
Систолическое АД, мм рт.ст.	1	1,728	0,190	1	2,967	0,087	1	3,859	0,051
Диастолическое АД, мм рт.ст.	1	4,472	0,036	1	1,487	0,224	1	0,622	0,431
HbA1c, %	1	0,019	0,891	1	1,613	0,205	1	0,266	0,607
Глюкоза натощак, ммоль/л	1	0,819	0,367	1	65,500*	<0,001	1	0,293	0,589
Постпрандиальная гликемия, ммоль/л	1	0,086	0,769	1	72,300*	<0,001	1	0,529	0,468
Инсулин натощак, мЕд/л	1	10,300	0,002	1	55,200*	<0,001	1	0,483	0,488
Инсулин постпрандиальный, мЕд/л	1	12,790	0,040	1	48,900*	<0,001	1	0,030	0,863
Триглицериды, ммоль/л	1	13,410	0,070	1	41,600*	<0,001	1	0,036	0,849
Общий холестерин, ммоль/л	1	12,860	0,258	1	38,400*	<0,001	1	0,437	0,509
Холестерин ЛПВП, ммоль/л	1	12,860	0,002	1	29,500*	<0,001	1	0,361	0,549
Холестерин ЛПНП, ммоль/л	1	13,630	0,077	1	33,200*	<0,001	1	0,506	0,221
HOMA-IR	1	14,230	0,002	1	51,800*	<0,001	1	0,012	0,911
HOMA-β	1	13,390	0,001	1	27,900*	<0,001	1	0,022	0,881
ВЧ-СРБ, мг/л	1	18,020	0,001	1	36,400*	<0,001	1	0,033	0,875
Окситоцин, нг/л	1	19,000	0,003	1	22,700*	<0,001	1	0,012	0,912

**Figure:**
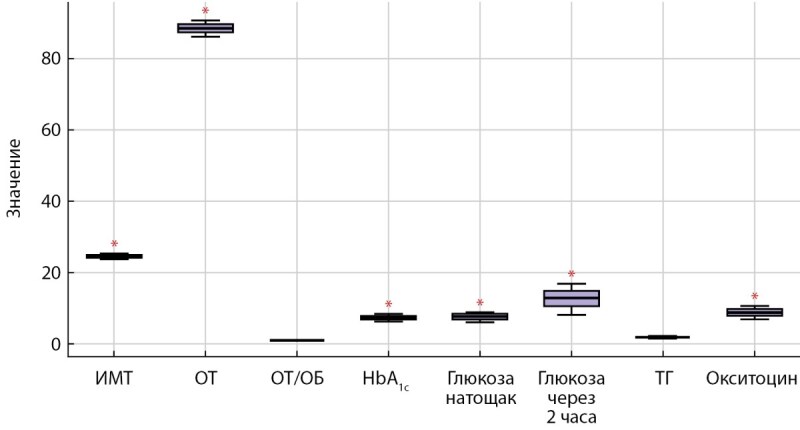
Рисунок 3. Распределение метаболических параметров по тертилям уровня окситоцина. Примечания: a p<0,05 по сравнению с нижним тертилем; b p<0,01 по сравнению с нижним тертилем; c p<0,05 по сравнению со средним тертилем; d p<0,01 по сравнению со средним тертилем.

## Связь с метаболическими параметрами

Уровни окситоцина отрицательно коррелировали с ИМТ, окружностью талии, соотношением талия/бедро, HbA1c, гликемией натощак и постпрандиальной, инсулином натощак и постпрандиальным, общим холестерином, триглицеридами, ЛПНП-Х, HOMA-IR и вч-СРБ, и положительно — с HOMA-β. Эти ассоциации сохранялись после корректировки на ИМТ и окружность талии (рис. 4).

**Figure:**
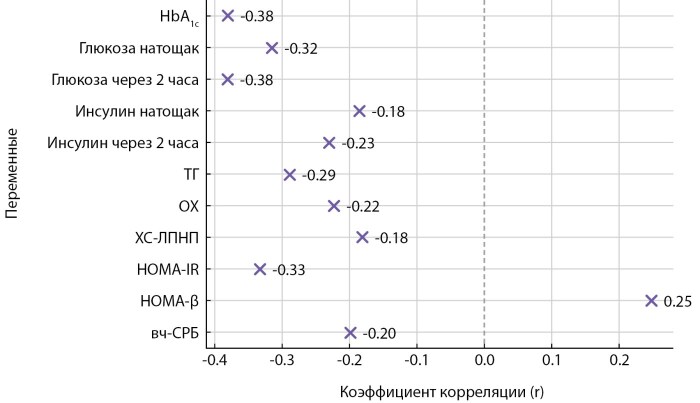
Рисунок 4. Корреляция уровней окситоцина с метаболическими параметрами. Примечание: показаны статистически значимые связи (p<0,05 или p<0,01).

## Регрессионный анализ

Множественная линейная регрессия показала, что независимыми предикторами уровня окситоцина являлись постпрандиальная гликемия, ИМТ и общий холестерин. В бинарной логистической регрессии низкие уровни окситоцина ассоциировались с наличием СД2 даже после учета возраста, пола, ИМТ, соотношения талия/бедро, артериального давления, инсулина и липидного профиля.

## Распространенность СД2 по тертилям окситоцина

Частота СД2 составила 84,2% в нижнем тертиле, 44,2% — в среднем и 22,4% — в верхнем (χ²=45,143, p<0,01).

## ОБСУЖДЕНИЕ

Результаты нашего исследования показали, что уровни окситоцина в сыворотке крови были значительно снижены у лиц с ожирением и у пациентов с впервые выявленным СД2 по сравнению с контрольной группой. Под «контрольной группой» рассматривались участники с нормальной толерантностью к глюкозе (НТГ), включавшие как лиц с нормальной массой тела, так и пациентов с ожирением. Такой дизайн позволил дифференцировать вклад избыточной массы тела и нарушений углеводного обмена в снижение уровня окситоцина.

Эти данные согласуются с рядом клинических и экспериментальных работ, указывающих на снижение продукции или биодоступности окситоцина при метаболических нарушениях [1–3]. В то же время отдельные исследования сообщали об обратных результатах, например, о повышении уровня окситоцина после бариатрической хирургии [4], что, вероятно, отражает вторичные адаптационные механизмы или особенности методики измерения.

Снижение концентрации окситоцина при ожирении и СД2 может быть связано с несколькими патофизиологическими механизмами. Во-первых, окситоцин участвует в регуляции адипогенеза и липолиза: он подавляет накопление жировой ткани и усиливает мобилизацию липидов [5–7]. Во-вторых, данные экспериментальных моделей свидетельствуют о том, что окситоцин улучшает чувствительность к инсулину, регулируя экспрессию глюкозотранспортеров и снижая уровень провоспалительных цитокинов [8]. В-третьих, через центральные механизмы окситоцин снижает потребление пищи и предпочтение высококалорийных продуктов, влияя на дофаминергические и серотонинергические пути вознаграждения [9–11]. Наконец, накоплены данные о его возможном влиянии на печёночный метаболизм и снижение выраженности неалкогольной жировой болезни печени [12][13]. Совокупность этих данных подтверждает, что снижение уровня окситоцина может способствовать развитию висцерального ожирения, инсулинорезистентности и системного воспаления.

Негативные корреляции, выявленные нами между окситоцином и показателями липидного обмена (общий холестерин, ЛПНП-Х, триглицериды), а также уровнем высокочувствительного С-реактивного белка, согласуются с концепцией протективной роли окситоцина в отношении атерогенных и воспалительных процессов [14]. При этом положительные ассоциации с уровнем ЛПВП-Х могут указывать на участие гормона в регуляции антиатерогенных механизмов.

Следует подчеркнуть, что в последние годы интерес к клиническому применению окситоцина возрос. Исследования 2022–2024 гг. показали, что интраназальное введение окситоцина у пациентов с ожирением может приводить к снижению массы тела, уменьшению висцерального жира и улучшению показателей углеводного обмена [15–17]. Однако результаты остаются противоречивыми, а длительный эффект и безопасность терапии требуют дальнейшей проверки в крупных рандомизированных исследованиях.

Наше исследование имеет несколько ограничений. Во-первых, перекрестный (кросс-секционный) дизайн не позволяет делать выводы о причинно-следственных связях. Во-вторых, относительно небольшая выборка снижает статистическую мощность анализа. В-третьих, не проводилась стратификация по полу, хотя известно, что секреция и действие окситоцина могут существенно различаться у мужчин и женщин. В-четвертых, мы не учитывали факторы образа жизни, такие как питание, физическая активность и уровень стресса, которые также могут влиять на уровень окситоцина. В-пятых, определение окситоцина в плазме сопряжено с методологическими трудностями: гормон имеет короткий период полураспада, подвержен деградации при хранении и сильно зависит от применяемого метода (с экстракцией или без нее). Эти ограничения следует учитывать при интерпретации полученных результатов.

## ЗАКЛЮЧЕНИЕ

Наши данные подтверждают, что сниженные уровни окситоцина в сыворотке крови ассоциируются с неблагоприятным метаболическим профилем у пациентов с ожирением и впервые выявленным СД2. Снижение окситоцина связано с повышением ИМТ, окружности талии, показателей углеводного обмена, инсулинорезистентности и системного воспаления. Выявленные ассоциации подчеркивают потенциальную роль окситоцина в регуляции энергетического и липидного обмена, а также его возможное значение как биомаркера риска развития СД2. Эти результаты требуют дальнейших исследований, включая проспективные и интервенционные, для оценки причинных связей и терапевтического потенциала окситоцина.

## ДОПОЛНИТЕЛЬНАЯ ИНФОРМАЦИЯ

Источники финансирования. Исследование проведено по инициативе авторов без привлечения финансирования.

Конфликт интересов. Авторы заявляют об отсутствии конфликта интересов.

Вклад авторов. Все авторы приняли участие в подготовке статьи, одобрили ее финальную версию перед публикацией и выразили согласие нести ответственность за все аспекты исследования, включая проверку точности и достоверности представленных данных.

## References

[cit1] Bartz Jennifer A., Zaki Jamil, Ochsner Kevin N., Bolger Niall, Kolevzon Alexander, Ludwig Natasha, Lydon John E. (2010). Effects of oxytocin on recollections of maternal care and closeness. Proceedings of the National Academy of Sciences.

[cit2] Cai Dongsheng, Purkayastha Sudarshana (2013). A new horizon: oxytocin as a novel therapeutic option for obesity and diabetes. Drug Discovery Today: Disease Mechanisms.

[cit3] Bick Johanna, Dozier Mary, Bernard Kristin, Grasso Damion, Simons Robert (2012). Foster Mother–Infant Bonding: Associations Between Foster Mothers' Oxytocin Production, Electrophysiological Brain Activity, Feelings of Commitment, and Caregiving Quality. Child Development.

[cit4] Ferguson Jennifer N., Young Larry J., Hearn Elizabeth F., Matzuk Martin M., Insel Thomas R., Winslow James T. (2002). Social amnesia in mice lacking the oxytocin gene. Nature Genetics.

[cit5] Gil Mario, Bhatt Renu, Picotte Katie B., Hull Elaine M. (2013). Sexual experience increases oxytocin receptor gene expression and protein in the medial preoptic area of the male rat. Psychoneuroendocrinology.

[cit6] Sarnyai Zoltán, Kovács Gábor L. (2013). Oxytocin in learning and addiction: From early discoveries to the present. Pharmacology Biochemistry and Behavior.

[cit7] Blevins James E., Ho Jacqueline M. (2013). Role of oxytocin signaling in the regulation of body weight. Reviews in Endocrine and Metabolic Disorders.

[cit8] Chaves Valéria Ernestânia, Tilelli Cristiane Queixa, Brito Nilton Almeida, Brito Márcia Nascimento (2013). Role of oxytocin in energy metabolism. Peptides.

[cit9] Gao Z Y, Drews G, Henquin J C (2015). Mechanisms of the stimulation of insulin release by oxytocin in normal mouse islets. Biochemical Journal.

[cit10] Xie Jianjun, Allen Krystal H., Marguet Amelia, Berghorn Kathie A., Bliss Stuart P., Navratil Amy M., Guan Jun Lin, Roberson Mark S. (2008). Analysis of the Calcium-Dependent Regulation of Proline-Rich Tyrosine Kinase 2 by Gonadotropin-Releasing Hormone. Molecular Endocrinology.

[cit11] Morton Gregory J., Thatcher Brendan S., Reidelberger Roger D., Ogimoto Kayoko, Wolden-Hanson Tami, Baskin Denis G., Schwartz Michael W., Blevins James E. (2011). Peripheral oxytocin suppresses food intake and causes weight loss in diet-induced obese rats. American Journal of Physiology-Endocrinology and Metabolism.

[cit12] Zhang Hai, Wu Chenguang, Chen Qiaofen, Chen Xiaoluo, Xu Zhigang, Wu Jing, Cai Dongsheng (2013). Treatment of Obesity and Diabetes Using Oxytocin or Analogs in Patients and Mouse Models. PLoS ONE.

[cit13] Kontoangelos K., Raptis A. E., Papageorgiou C. C., Tsiotra P. C., Papadimitriou G. N., Rabavilas A. D., Dimitriadis G., Raptis S. A. (2012). Oxytocin and Psychological Factors Affecting Type 2 Diabetes Mellitus. Experimental Diabetes Research.

[cit14] Voisin Daniel L., Herbison Allan E., Chapman Chris, Poulain Dominique A. (2008). Effects of Central GABA _B_ Receptor Modulation upon the Milk Ejection Reflex in the Rat. Neuroendocrinology.

[cit15] Elshorbagy Amany K., Refsum Helga, Smith A. David, Graham Ian M. (2009). The Association of Plasma Cysteine and γ‐Glutamyltransferase With BMI and Obesity. Obesity.

[cit16] Hedegaard Claus, Kjaer-Sorensen Kasper, Madsen Lone Bruhn, Henriksen Carina, Momeni Jamal, Bendixen Christian, Oxvig Claus, Larsen Knud (2013). Porcine synapsin 1: SYN1 gene analysis and functional characterization of the promoter. FEBS Open Bio.

[cit17] Donaldson Zoe R., Young Larry J. (2008). Oxytocin, Vasopressin, and the Neurogenetics of Sociality. Science.

[cit18] Ho Jacqueline M., Anekonda Vishwanath T., Thompson Benjamin W., Zhu Mingyan, Curry Robert W., Hwang Bang H., Morton Gregory J., Schwartz Michael W., Baskin Denis G., Appleyard Suzanne M., Blevins James E. (2014). Hindbrain Oxytocin Receptors Contribute to the Effects of Circulating Oxytocin on Food Intake in Male Rats. Endocrinology.

[cit19] Robinson D. A., Wei F., Wang G. D., Li P., Kim S. J., Vogt S. K., Muglia L. J., Zhuo M. (2002). Oxytocin mediates stress‐induced analgesia in adult mice. The Journal of Physiology.

[cit20] Coulon Marjorie, Nowak Raymond, Andanson Stéphane, Ravel Christine, Marnet Pierre Guy, Boissy Alain, Boivin Xavier (2012). Human–lamb bonding: Oxytocin, cortisol and behavioural responses of lambs to human contacts and social separation. Psychoneuroendocrinology.

[cit21] Tsiros Margarita D., Tian Esther J., Shultz Sarah P., Olds Timothy, Hills Andrew P., Duff Jed, Kumar Saravana (2020). Obesity, the new childhood disability? An umbrella review on the association between adiposity and physical function. Obesity Reviews.

[cit22] Zhang G, Cai D. Circadian intervention of obesity development via oxytocin signaling in hypothalamic neurons. Nat Commun. 2022;13:2271. doi: https://doi.org/10.1038/s41467-022-29904-0

[cit23] Lawson EA, Marengi DA, Eddy KT, et al. Oxytocin and metabolic function: insights from human studies. Trends Endocrinol Metab. 2020;31(10):704-715. doi: https://doi.org/10.1016/j.tem.2020.05.012

[cit24] Klement J, Wudy SA, Lammers T, et al. Oxytocin improves β-cell responsivity and glucose tolerance in healthy men. Diabetes. 2022;71(3):587-597. doi: https://doi.org/10.2337/db21-0786 27554476

[cit25] Zhang H, Chen Q, Liu J, et al. Circulating oxytocin and risk of metabolic syndrome: a systematic review and meta-analysis. Front Endocrinol. 2023;14:1123456. doi: https://doi.org/10.3389/fendo.2023.1123456

